# Association of serum chloride concentration with outcomes in postoperative critically ill patients: a retrospective observational study

**DOI:** 10.1186/2052-0492-2-39

**Published:** 2014-06-23

**Authors:** Satoshi Kimura, Shinsaku Matsumoto, Nagisa Muto, Tomoko Yamanoi, Tatsuya Higashi, Kosuke Nakamura, Mineo Miyazaki, Moritoki Egi

**Affiliations:** Department of Anesthesiology, Kure Kyosai Hospital, 2-3-28, Nishichuo, Kure City, Hiroshima, 737-8505 Japan; Department of Anesthesiology, Kobe University Hospital, 7-5-2, Kusunoki cho, Chuo-ku, Kobe City, Hyogo, 650-0017 Japan

**Keywords:** Hypochloremia, Critically ill, Mortality

## Abstract

**Background:**

Although chloride is one of the major electrolytes measured routinely in dairy practice, the amount of attention chloride receives in critically ill patients is limited. There are still a few studies reporting the incidence of derangements of chloride and its association with patients' outcomes. Accordingly, we conducted a retrospective study to assess the prevalence of abnormality of serum chloride level in postoperative patients in the intensive care unit on the early phase of surgery and its association with outcome.

**Methods:**

We conducted a single-center retrospective observational study. All adult patients who underwent elective thoracic or abdominal surgery and required postoperative intensive care for more than 48 h between 2007 and 2011 were included. Chloride levels were measured on each morning of postoperative day 1 and day 2 in the intensive care unit. We defined all-cause hospital death as the primary outcome and compared serum chloride levels on postoperative day 1 and day 2 between hospital survivors and non-survivors. Comparisons among groups were conducted using the chi-square test for equal proportion, Mann-Whitney *U* tests, or Kruskal-Wallis test.

**Results:**

Among 98 patients included in this study, hypochloremia (less than 98 mmol/L) during the first 48 h occurred in 14 patients (14.3%). The mortality in hypochloremia patients was 28.6%, which is significantly higher than 6.0% in patients with normal chloride concentration (*p* = 0.007). Even after being adjusted for severity of illness, the incidence of hypochloremia was independently associated with the risk of hospital death (adjusted odds ratio 5.8 (1.1, 30.2), *p* = 0.04). Hyperchloremia (more than 112 mmol/L) occurred in one patient (1.0%), who was discharged from the hospital at day 9. There was no significant difference in the total volume of infused fluid (*p* = 0.30), sum of chloride administration (*p* = 0.33), and use of furosemide (*p* = 0.75) from intensive care unit admission to the morning of postoperative day 2 between survivors and non-survivors.

**Conclusions:**

Hypochloremia observed within 48 h after surgery was not rare and was independently associated with the increased risk of hospital death. Hypochloremia might be a useful indicator of prognosis for patients in the postoperative intensive care unit.

## Background

Derangements of serum sodium concentration in critically ill patients are common and appear to be associated with worse outcomes [1–3]. Chloride is the most abundant anion in plasma and interstitial fluid, accounting for approximately one third of plasma tonicity, and affects acid-base disorder in the light of physicochemical approach [4]. Although chloride is one of the major electrolytes measured routinely in dairy practice, the amount of attention chloride receives in critically illness is limited [5]. There are still a few studies reporting the incidence of derangements of chloride and its association with patients' outcomes. Accordingly, we conducted a retrospective study to assess the prevalence of abnormality of serum chloride level in postoperative intensive care unit (ICU) patients on the early phase of surgery and its association with outcome. Additionally, we compared these associations with those of sodium concentrations.

## Methods

### Study design

We conducted a single-center retrospective observational study. The Kure Kyosai Hospital Ethics Committee approved this investigation. The committee waived the need for informed consent for studies involving the use of the database.

### Patients

All adult patients who underwent elective thoracic or abdominal surgery and required postoperative intensive care for more than 48 h between 2007 and 2011 were included in this study. We excluded patients with missing relevant values: the choroid concentration and the type and amount of fluid given intravenously.

### Primary outcome

The primary outcome of this analysis was all-cause hospital mortality, defined as death before hospital discharge.

### Data source

The serum chloride data used for this study were stored and retrieved electronically. Age, sex, requirement for mechanical ventilation, category of surgery, operative duration, operative blood loss, Acute Physiology and Chronic Health Evaluation (APACHE) II score [6], and information on intravenous fluid administration were obtained from the clinical database.

### Serum chloride and sodium concentrations and blood gas analysis

Serum chloride and sodium concentrations were measured in all patients prior to surgery as baseline and on each morning of postoperative days (PODs) 1 and 2. Serum chloride and sodium concentrations were measured by an ion-selective electrode (C8000/200FR, Toshiba, Tochigi, Japan). According to the normal chloride concentration of our machine (98–112 mmol/L), hypochloremia and hyperchloremia were defined as Cl concentrations less than 98 mmol/L and more than 112 mmol/L, respectively. We also obtained arterial pH, PaCO_2_, and bicarbonate simultaneously measured with chloride concentration.

### Statistical analysis

Data are presented as percentages (*n*) or as median (25% quartile, 75% quartile), since the continuous data used in this study were not normally distributed. Comparisons among groups were conducted using the chi-square test for equal proportion, Mann-Whitney *U* tests, or Kruskal-Wallis test. The correlations of two values were assessed using Pearson's correlation coefficient.

We first separated the patients into hospital survivors and non-survivors to assess the possible confounders of death. To determine the independent contribution of hypochloremia and hyperchloremia to the prediction of hospital death, we constructed multivariate models using potential predictors of death (criteria for inclusion at *p* = 0.2). Results from the multivariate models are shown using odds ratios with 95% confidence intervals (CIs). All statistical analyses were performed using a commercially available statistical software (SPSS 19.0; SPSS, Inc., Chicago, IL, USA). Data were reported in accordance with the Strengthening the Reporting of Observational Studies in Epidemiology guidelines [7].

## Results

**Figure 1:**
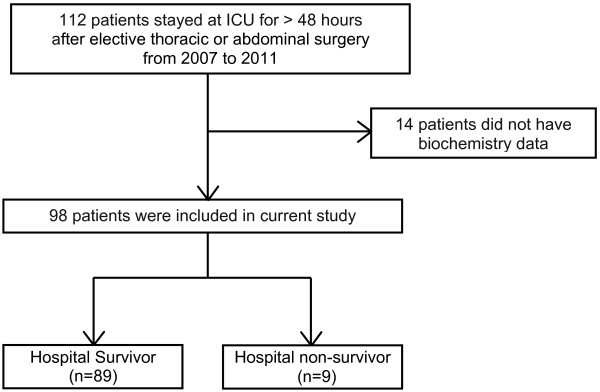
**Study flow.**

During the study period, there were 112 patients who required intensive care postoperatively for more than 48 h. Fourteen patients were excluded because of lack of biochemistry data or information on intravenous fluid administration. Thus, 98 patients were eligible for the study and were included in the final analysis. All patients were followed up until discharge or death (Figure 1).

The mean serum chloride concentration on POD 1 was 104 (102, 106) mmol/L, which was not significantly different from the preoperative value 104 (102, 106) mmol/L (*p* = 0.92). The mean serum chloride concentration on POD 2 was 102 (100, 105) mmol/L, which was significantly lower than the preoperative value (*p* = 0.002) and that on POD 1 (*p* < 0.0001).

The mean serum sodium concentration on POD 1 and POD 2 was 137 (135, 140) and 137 (134, 139) mmol/L, respectively, which was significantly lower than the preoperative sodium concentration 140 (137, 142) mmol/L (*p* < 0.0001). The mean arterial pH on POD 2 was 7.44 (7.42, 7.46), which was significantly higher than 7.43 (7.40, 7.45) on POD 1 (*p* < 0.0001). The mean base excess on POD 2 was 1.6 (0.4, 2.9), which was significantly higher than 0.3 (-1.1, 1.5) on POD 1 (*p* < 0.0001).

Among these 98 patients, 9 patients died in the hospital (hospital mortality 9.2%). Table 1 shows the comparison of patient demographics between hospital survivors and non-survivors. Survivors were significantly less sick than non-survivors (APACHE II 11 vs. 14, *p* = 0.03).

**Table 1:** **Comparison of patient demographics between survivors and non-survivors**

Characteristics	Survivors (***n*** = 89)	Non-survivors (***n*** = 9)	***p***value
Age, years (IQR)	73 (67, 80)	76 (72, 83)	0.19
Male, *n* (%)	57 (64)	6 (67)	0.88
Mechanical ventilation, *n* (%)	25 (28)	4 (44)	0.31
Operative duration, minute (IQR)	380 (265, 525)	435 (183, 670)	0.78
Blood loss, mL (IQR)	620 (375, 1,355)	500 (310, 3,900)	0.89
Thoracic surgery, *n* (%)	6 (7)	1 (11)	0.63
Abdominal surgery, *n* (%)	83 (93)	8 (89)	0.63
ICU stay, days (IQR)	4 (3, 6)	5 (3, 20)	0.24
APACHE II (IQR)	11 (9, 14)	14 (12, 20)	0.03

*ICU* intensive care unit, *APACHE* Acute Physiology and Chronic Health Evaluation, *IQR* interquartile range.

Within the first 48 h in ICU, there are 14 patients (14.3%) who showed at least one hypochloremia. Hypochloremia occurred in 5 patients (5.1%) on POD 1 and in 14 patients (14.3%) on POD 2. The mortality in hypochloremia patients was 28.6%, which is significantly higher than 6.0% in patients with normal chloride concentration (*p* = 0.007). Hyperchloremia occurred in one (1.0%) patient on POD 1. The patient with hyperchloremia was discharged from the hospital on POD 9.

Table 2 shows the comparison of serum chloride concentration between survivors and non-survivors. The serum chloride concentration of non-survivors on POD 1 and POD 2 was significantly lower than that of survivors (98 (96, 104) vs. 104 (102, 106), *p* = 0.02; 100 (95, 103) vs. 103 (100, 105), *p* = 0.03, respectively). Table 2 also shows the comparison of intravenous fluid administration (crystalloid and colloid) from ICU admission to the morning of POD 2 between survivors and non-survivors. There was no significant difference in the total volume of fluid (*p* = 0.30), sum of chloride administration (*p* = 0.33), and use of furosemide (*p* = 0.75).

**Table 2:** **Comparison of serum chloride concentration and chloride administration between survivors and non-survivors**

	Survivors (***n*** = 89)	Non-survivors (***n*** = 9)	***p***value
Cl on Pre-POD, meq/L (IQR)	104 (102, 106)	100 (95, 106)	0.06
Cl on POD 1, meq/L (IQR)	104 (102, 106)	98 (96, 104)	0.02
Cl on POD 2, meq/L (IQR)	103 (100, 105)	100 (95, 103)	0.03
Total volume of intravenous fluid administration, mL (IQR)	4,175 (3,660, 4,875)	4,180 (2,070, 5,141)	0.30
Sum of intravenous chloride administration, meq (IQR)	314 (246, 386)	289 (113, 476)	0.33
Average chloride concentration in intravenous fluid, meq/L (IQR)	76 (67, 83)	69 (53, 93)	0.61
Use of furosemide, *n* (%)	16 (18)	2 (22)	0.75

*Pre-POD* pre-operation, *POD* postoperative day, *Cl* serum chloride concentration, *IQR* interquartile range.

Table 3 shows the comparison of serum sodium concentration, lactate concentration, arterial pH, PaCO_2_, and bicarbonate concentration simultaneously measured with postoperative chloride concentration between survivors and non-survivors. There was no significant difference in them between survivors and non-survivors.

**Table 3:** **Comparison of serum sodium concentration, arterial pH, PaCO**_**2**_ **, and bicarbonate between survivors and non-survivors**

	Survivors (***n*** = 89)	Non-survivors (***n*** = 9)	***p***value
Na on POD 1, meq/L (IQR)	137 (136, 140)	134 (130, 141)	0.14
Na on POD 2, meq/L (IQR)	137 (134, 139)	135 (128, 137)	0.10
Arterial pH on POD 1	7.42 (7.40, 7.44)	7.43 (7.39, 7.47)	0.42
Arterial pH on POD 2	7.44 (7.41, 7.46)	7.46 (7.3, 7.48)	0.08
PaCO_2_ on POD 1, mmHg (IQR)	37.5 (35.3, 40.8)	34.2 (33.6, 39.0)	0.20
PaCO_2_ on POD 2, mmHg (IQR)	38.6 (36.0, 41.8)	36.8 (33.9, 38.7)	0.19
Bicarbonate on POD 1, mmol/L(IQR)	24.4 (23.1, 25.7)	24.6 (20.9, 25.9)	0.86
Bicarbonate on POD 2, mmol/L (IQR)	25.5 (24.1, 26.9)	25.8 (24.7, 26.5)	0.69

*POD* postoperative day, *Na* serum sodium concentration, *IQR* interquartile range.

As higher severity of illness confounded the possible association between hypochloremia and higher mortality, we performed multivariate logistic analysis. Even after being adjusted for APACHE II score, the incidence of hypochloremia was independently associated with the risk of hospital death (adjusted odds ratio 5.8, 95% CI (1.1, 30.2), *p* = 0.04) (Table 4).

**Table 4:** **Multivariable analysis for hospital death**

Variables	Odds ratio (95% CI)	***p***value
APACHE II	1.3 (1.1, 1.7)	0.006
Hypochloremia (vs. normo-chloremia)	5.8 (1.1, 30.2)	0.04

*APACHE* Acute Physiology and Chronic Health Evaluation, *CI* confidence interval.

## Discussion

### Key results

In our retrospective analysis with 98 postoperative patients, hypochloremia within postoperative 48 h in ICU was not rare (14.3%). Patients with postoperative hypochloremia had a significantly higher mortality compared with those with normal chloride levels. Even after being adjusted for severity of illness, the incidence of hypochloremia was independently associated with the risk of hospital death. In the current cohort, the incidence of hyperchloremia was not common; thus, its association with outcomes could not be assessed.

### Comparison with prior publication

So far, there are two studies reporting the prevalence of hypochloremia and its association with outcomes in critical illness. Terzano et al. showed that a lower serum chloride concentration was associated with a longer duration of non-invasive mechanical ventilation in patients with chronic obstructive pulmonary disease exacerbation [8]. They showed that its duration was significantly associated with lower sodium concentration and lower arterial pH as well. They have not defined the definition of hypochloremia, so its prevalence was not reported. In this study, there was no report on the cause of hypochloremia.

Tani et al. reported that the incidence of hypochloremia (<98 mmol/L) in mixed ICU patients was 8.8%. They concluded that patients with hypochloremia had a significantly longer ICU stay and higher hospital mortality than those with normal chloride concentration and hyperchloremia [9]. They showed that lower sodium, higher potassium, and lower bicarbonate concentrations and lower PaCO_2_ were also significantly associated with increased mortality. The cause of derangement of chloride concentrations was not reported in this study.

Our finding in this study gave close agreement with that in the study of Tani et al. We believe that this is the first study assessing the *independent* association of hypochloremia with outcomes in ICU patients. In this regard, this study might add novel information on prior findings.

There are several studies reporting the prevalence of hyperchloremia and its association with outcomes [10, 11]. In our study, postoperative hyperchloremia was recognized in only one patient (1%). Thus, it is impossible to determine its association with outcomes. Such a variation of prevalence and association with outcomes would be due to the different case mix and patient care. To confirm this fact, a further larger study with more detailed information should be necessary.

### Limitations

There were several limitations in this study. First, this was an observational study in nature, and thus, our findings showed an association but not a causality link. Second, this is retrospective in design and thus potentially subject to systematic error and bias. However, the discharge status was objective, and the electronic data were numerical in nature and measured independently. Thus, they were not amenable to unintended manipulation. Third, as this study was conducted in surgical ICU, our findings could not be generalized into non-surgical ICU patients. Similar studies for internal ICU patients need to be conducted in the near future.

Finally, our study was conducted for only 48 h postoperatively, which might be too short to observe the development of postoperative electrolyte derangements. Thus, observation for a longer period should be conducted in a future study. However, we believe that this study would still be important, because it showed that hypochloremia seen in the early stage of the postoperative period was independently associated with mortality.

### The cause of hypochloremia

Chloride is mainly regulated by the intestinal duct and kidney [12]. In the stomach, chloride is secreted as a gastric fluid and enteric fluid and then reabsorbed throughout the intestinal duct. In the kidney, chloride is filtered at the glomeruli, and more than 99% is reabsorbed at the distal tubule. Thus, the major cause of hypochloremia in critical illness was considered to be loss of chloride, which could be caused by the use of diuretics, loss of gastric fluid or adrenal insufficiency, and overvolume of water.

As the current study as well as prior studies [8, 9] is a retrospective observational study, the cause of hypochloremia was not ascertained [8, 9]. In our patients, we expect that one cause of hypochloremia was loss of gastric juice because we mainly included patients undergoing abdominal surgery, most of whom was put on a gastric tube during and after surgery. Another cause of abnormality of chloride concentration might be the derangement of adrenal hormones triggered by surgical stress.

### The association of hypochloremia with increased mortality

There are some potential mechanisms that explain the relation between hypochloremia and increased mortality.

First, hypochloremia would be a sign of complicated surgery, which may lead to larger surgical stress. It might cause more loss of gastrointestinal tract fluid or relative derangement of adrenal hormones, thus would lead to hypochloremia [13]. These conditions might likely contribute to the association between hypochloremia and mortality. Unfortunately, the current study could not assess the impact of surgical stress on the relationship between hypochloremia and mortality. In this regard, a future prospective study collecting information on the postoperative adrenal function, inflammatory response, and loss of chloride from the kidney should be conducted to refute or confirm this speculation.

Second, hypochloremia would be a sign of the severity of illness, as an association of hypochloremia with higher mortality existed with the absence of significant differences in intravenous chloride administration and acid-base status. It is no wonder for critically ill patients with various commodities to have poor regulation of homeostasis in the body including serum chloride concentration. If it is true, one might be able to use early postoperative serum chloride concentration for prediction of the worse outcomes. Finally, there might be any combination of the above mechanisms.

We should note that this study is a hypothesis-generating study promoting a future prospective study, which may assess the cause of hypochloremia and the mechanism of its association with worse outcomes.

We also note that our results showed just the association of hypochloremia and higher mortality, but not the causality link. Yunos et al. recently showed that a chloride-restrictive intravenous fluid strategy in ICU improved patients' outcomes [14]. In this regard, our finding suggested that hypochloremia could be used as one of the markers of severity and predictors of worse outcomes, but not the therapeutic target.

## Conclusions

In conclusion, hypochloremia observed within 48 h after surgery was not rare and was independently associated with the increased risk of hospital death. Hypochloremia might be a useful indicator of prognosis for postoperative ICU patients.

## Abbreviations

APACHE: Acute Physiology and Chronic Health Evaluation
CI: confidence interval
ICU: intensive care unit
POD: postoperative day.
